# Supplementary material for: The causal role of circulating inflammatory markers in osteoporosis: a bidirectional Mendelian randomized study
Source: Front Immunol. 2024 Jul 18;15:1412298. doi: 10.3389/fimmu.2024.1412298 (PMC11291241; doi:10.3389/fimmu.2024.1412298)
Supplement: Supplementary file 1 [file DataSheet_1.zip › supplementary material/OSTEOPOROSIS-CIM/GCST90274814.tsv.gz_forest.pdf]

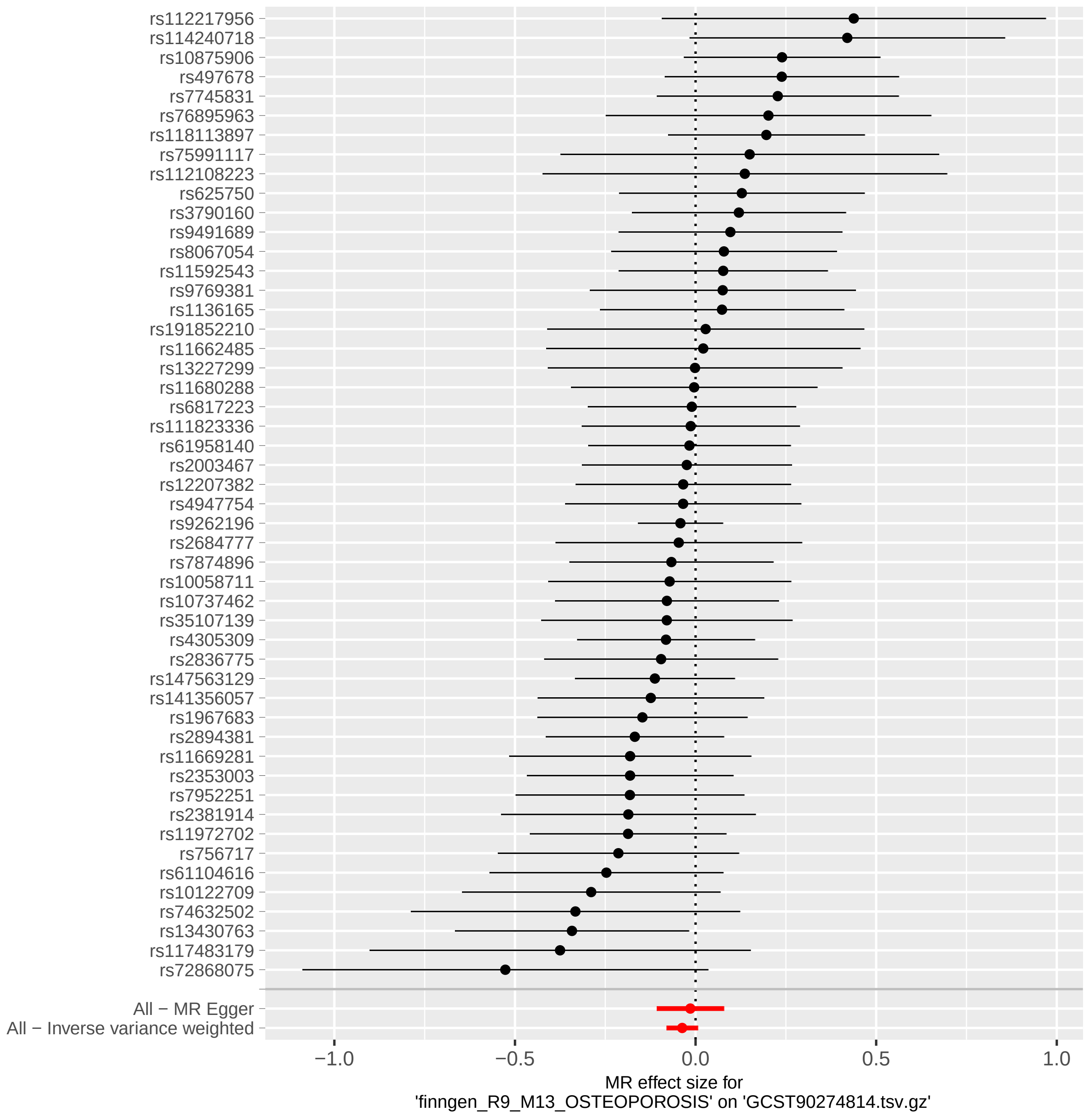
rs112217956
rs114240718
rs10875906
rs497678
rs7745831
rs76895963
rs118113897
rs75991117
rs112108223
rs625750
rs3790160
rs9491689
rs8067054
rs11592543
rs9769381
rs1136165
rs191852210
rs11662485
rs13227299
rs11680288
rs6817223
rs111823336
rs61958140
rs2003467
rs12207382
rs4947754
rs9262196
rs2684777
rs7874896
rs10058711
rs10737462
rs35107139
rs4305309
rs2836775
rs147563129
rs141356057
rs1967683
rs2894381
rs11669281
rs2353003
rs7952251
rs2381914
rs11972702
rs756717
rs61104616
rs10122709
rs74632502
rs13430763
rs117483179
rs72868075
All − MR Egger
All − Inverse variance weighted
−1.0
−0.5
0.0
0.5
1.0
MR effect size for
'finngen_R9_M13_OSTEOPOROSIS' on 'GCST90274814.tsv.gz'
